# N-acetylcysteine for cessation of tobacco smoking: rationale and study protocol for a randomised controlled trial

**DOI:** 10.1186/s13063-019-3628-5

**Published:** 2019-09-10

**Authors:** Lauren Arancini, Chiara C. Bortolasci, Seetal Dodd, Olivia M. Dean, Michael Berk

**Affiliations:** 1 IMPACT Strategic Resource Centre, School of Medicine, Deakin University, P.O. Box 281, Geelong, VIC 3220 Australia; 2 Centre for Molecular and Medical Research, School of Medicine, Deakin University, Geelong, VIC Australia; 3 Barwon Health, University Hospital Geelong, Geelong, VIC Australia; 4 Department of Psychiatry, The University of Melbourne, Parkville, VIC Australia; 5 Orygen, the National Centre for Excellence in Youth Mental Health, Parkville, VIC Australia; 6 Florey Institute of Neuroscience and Mental Health, Parkville, VIC Australia

**Keywords:** N-acetylcysteine, Smoking cessation, Nicotine, Intervention, Protocol

## Abstract

**Background:**

Tobacco smoking is a highly prevalent, addictive behaviour and a key public health priority. However available cessation therapies have low quit and high relapse rates, indicating an urgent need for more effective treatments. Predicated on promising preclinical and pilot clinical data, this paper presents a rationale and protocol for the trial of N-acetylcysteine (NAC) as a novel anti-craving smoking cessation aid.

**Methods:**

Current smokers (*n* = 120) of at least 10 cigarettes a day are recruited through online advertisements, print publications and dissemination of flyers. Participants are randomised on a 1:1 ratio to receive either 16-week treatment of 1.8 g/day of NAC or placebo with all participants receiving quit support from the online QuitCoach tool. Participants are attending visits at baseline, 8 and 16 weeks with a 42-week post-discontinuation follow-up. The primary outcome measure is sustained abstinence at six months after treatment based on self-reported rating scales and confirmed by exhaled carbon monoxide and salivary cotinine levels. Secondary outcomes are timing of the first lapse and relapse, between-group cigarette consumption, withdrawal symptoms, general wellbeing and mood/anxiety symptoms. Between-group differences in adverse events and subgroup analyses for variables including gender and Diagnostic Statistics Manual 5 diagnostics will also be investigated.

**Discussion:**

The planned trial addresses an issue of major importance to human health and, if an effect is shown, may result in substantial changes to the management of smoking and nicotine addiction with overt public health implications.

**Trial registration:**

Australian New Zealand Clinical Trials registry (ANZCTR), ACTRN12617001478303. Registered on 19 October 2017.

**Electronic supplementary material:**

The online version of this article (10.1186/s13063-019-3628-5) contains supplementary material, which is available to authorized users.

## Introduction

Tobacco smoking is the leading cause of preventable morbidity and mortality globally [1] and kills > 19,000 people in Australia each year [2], which includes between one-half and two-thirds of all long-term users [3]. While the prevalence rate in the general community has decreased over the past decade, linked to effective public health interventions, the rate of decrease in the prevalence of smoking has been slowing and there remains a substantial core group of persistent smokers.

It is essential to trial new strategies and medications to make further significant gains in reducing tobacco smoking. Developing a safer, better tolerated pharmacotherapy for smoking cessation is a critical step towards making further gains. Vulnerable groups and special populations may especially benefit from a low-cost, over-the-counter, effective pharmacotherapy with a better safety profile than existing pharmacotherapies such as varenicline and bupropion [4–8].

N-acetylcysteine (NAC) has antioxidant properties, both increasing glutathione and modulating glutamatergic, neurotropic, mitochondrial and inflammatory pathways [9]. NAC is a globally available nutraceutical supplement that is well tolerated, with a side effect profile that does not differ significantly from placebo in clinical trials when administered orally at doses up to 3 g/day with the exception of usually mild gastrointestinal side effects [10]. There are data to suggest that it is efficacious for reducing the number of cigarettes smoked [11] and relapse prevention [12]. NAC may also improve some of the physical harms caused by tobacco smoke exposure, improving mucociliary transport [13] and preventing oxidative damage to lung and other tissues [14, 15].

There are four small published pilot studies investigating the effects of NAC in nicotine dependence. A short-term abstinence study of heavy smokers (*n* = 22), treated for 3.5 days with NAC (3.6 g/day) or placebo, identified that NAC-treated participants reported that their first cigarette was significantly less rewarding when compared with the first cigarette of placebo-treated smokers [12]. A four-week, open-labelled study of tobacco smokers (*n* = 19) treated with NAC and varenicline reported a reduction in cigarettes smoked per day [16]. A randomised trial reports that after four weeks of 2.4 g/day of NAC treatment (*n* = 33, compared with placebo), there was a significant decrease in the number of cigarettes smoked, but no reduction in craving, withdrawal symptoms or level of carbon monoxide.

In another pilot trial, participants were randomised to 12 weeks of 3 g/day NAC (*n* = 17) or placebo (*n* = 17) adjunctive to addiction-focused cognitive behavioural therapy. NAC treatment significantly reduced the number of cigarettes smoked daily, exhaled carbon monoxide and improved depression scores. Quit rates were 47.1% in those treated with NAC versus 21.4% of placebo-treated patients. There were no significant differences in treatment emergent adverse events (AEs) between both groups. These pilot data are very promising [17] and suggest the need for larger more definitive trials of NAC for smoking cessation.

### Study aims and hypotheses

The aim of this study is to investigate the efficacy of NAC (1.8 g/day) for smoking cessation in a randomised, placebo-controlled trial of current smokers who wish to quit smoking. The primary outcome measure will be 24 weeks of continuous abstinence from end of treatment (EoT; 16 weeks) in tobacco smoking, confirmed by biological measures. Secondary outcome measures include point prevalence abstinence, time to relapse and total cigarette consumption. Safety, tolerability and subgroup analyses will also be conducted.

It is hypothesised that: (1) treatment with NAC will be superior to placebo for smoking cessation at follow-up (week 42), confirmed by assaying exhaled carbon monoxide, salivary cotinine (COT); and (2) treatment with NAC will be superior to placebo for smoking cessation at treatment endpoint (week 16), confirmed by assaying exhaled carbon monoxide, salivary COT.

## Methods

### Overview

This is a double-blind, randomised, placebo-controlled trial, to compare 1.8 g/day of NAC with placebo for smoking cessation, conducted over a period of 16 weeks. A six-month post-treatment discontinuation (week 42) follow-up is assessing ongoing abstinence. Data are collected by interview at baseline and weeks 8 and 16, and at the follow-up visit, six months after completion of the trial or 42 weeks in total. The trial has approval from the relevant research and ethics committees and is being conducted in accordance with the Good Clinical Practice (GCP), Australian Clinical Trial guidelines and the National Ethical guidelines for Human Research. The trial is registered on the Australian New Zealand Clinical Trials registry (ANZCTR), ACTRN12617001478303. This study was approved by the Barwon Health Human Research Ethics Committee (reference: 17/15).

The study is being conducted at two sites, Barwon Health, Geelong and the Deakin University Burwood campus. Participants (*n* = 120) are randomised to 16 weeks of treatment with NAC (1.8 g/day) or placebo effervescent tablets taken with water. The first participant was recruited in November 2017 and recruitment is ongoing. The study is being sponsored by Barwon Health and is supported by the ARC Harry Windsor Research Grants Scheme 2017.

### Inclusion and exclusion criteria

Participants are required to meet the following criteria: currently planning to quit tobacco smoking and set a quit date no more than one week from the proposed trial commencement date; aged ≥ 18 years; capacity to consent to the study and to follow its instructions and procedures; current daily smoker of ≥ 10 cigarettes per day. Participants are ineligible to enter the trial under the following conditions: known or suspected clinically unstable systemic medical or psychiatric disorders that require acute medical treatment; current use of oral glucocorticoids; active gastrointestinal ulcers; pregnancy or breastfeeding, or planning a pregnancy within six months; current use of > 500 mg NAC/day; a history of anaphylactic reaction to NAC or any component of the preparation. Only one member of a household is permitted to participate in the trial concurrently to minimise cohort-confounding effects. There are no restrictions on bupropion, varenicline or nicotine replacement therapy (NRT) use before the baseline visit. Female participants are required to be utilising effective contraception if of childbearing age and sexually active.

### Withdrawal criteria

Participants are withdrawn from the trial under the following conditions: failure to take the trial medication for seven consecutive days; pregnancy; emergence of serious AEs suspected to be associated with the trial medication; or commencement of a different pharmacotherapy for smoking cessation during the 16-week treatment phase. Participants will be questioned about these events and their adherence to the study protocol at the bi-weekly telephone interviews during the treatment phase of the study. Withdrawal of consent at any time in the study will result in immediate withdrawal from the trial.

### Recruitment

Recruitment is anticipated to occur mainly through targeted online advertising. Other recruitment techniques will include the use of flyers, which will be displayed in the waiting rooms of health services and other conspicuous locations. Print and radio advertisements will be included where possible. Following initial contact by potential participants, a researcher will establish that inclusion and non-inclusion criteria are satisfied. Written informed consent for the trial will be obtained from all participants at the first meeting.

### Trial procedure

All participants will provide informed written consent before they undergo a baseline face-to-face interview with a trained researcher. Participant contact will be conducted using procedures adapted from the treatment manual for the Healthy Lifestyles Program [18, 19]. Consented participants are randomised to receive NAC or placebo through a 1:1 allocation ratio and commenced on a standardised smoking cessation program using the automated QuitCoach website. The QuitCoach is a tailored, Internet-delivered smoking cessation advice program [20]. It is designed to replicate face-to-face multi-session smoking cessation counselling. QuitCoach has been shown to be well accepted by people who are planning to quit smoking and can be successfully combined with pharmacotherapy [21]. Participants will be randomly and sequentially allocated, in a double-blind fashion, to receive NAC or placebo from day 0 of the treatment phase of the study to the end of week 16. A fixed dose regime of 1.8 g/day of NAC, administered as one 900 mg effervescent tablet, is to be dissolved in a glass of water and consumed, twice daily. To facilitate the double-blinding process, the trial medications (both NAC and placebo) will be dispensed in identical numbers and tablet formulations in sealed containers by the trial pharmacist, who will also perform the tablet counts to monitor adherence. Participants will collect their medications from the trial clinician at baseline and at the eight-week study visit.

### Collection and handling of biological samples

Saliva will be collected at baseline and at weeks 8, 16 and 42 for COT measurements. Before the baseline meeting, participants will be provided with the following instructions: (1) avoid foods with high sugar or acidity, or high caffeine content immediately before sample collection; (2) do not eat a major meal within 60 min of sample collection; (3) do not consume alcohol within 12 h of sample collection; (4) participants should not brush their teeth within 45 min before sample collection; (5) dental work should not be performed within 24 h before sample collection. The trial clinician will document consumption of alcohol, nicotine, caffeine and medications in the 12 h before collection, as well as vigorous physical activity and the presence of oral disease or injury. Saliva collection is performed by instructing participants to allow saliva to pool in the mouth and then drool through the Salimetrics Saliva Collection Aid into a cryovial. This sample is stored in a – 80 °C freezer until it is time for analysis. An enzyme-linked immunosorbent assay (ELISA) will be performed to determine levels of COT in the samples. Samples are collected at each participant meeting.

### Allocation and concealment

Individuals will be assigned in a 1:1 ratio using permutated block randomisation to treatment with NAC or placebo, in a double-blind fashion. The randomisation code is generated by an independent researcher and held by the pharmacy to maintain blinding. The randomisation code remains concealed until all data analysis has been completed (triple-blind design). Unblinding for emergency situations is undertaken by the pharmacy, with the approval of the Principle Investigator.

### Treatment adherence and fidelity

The trial medications will be supplied by the trial pharmacist; participants will be instructed to return all containers to allow capsule counts. Concordant with SPIRIT guidelines, adherence will be assessed by pill counts of returned medication packs. Use of QuitCoach will be monitored by participant self-report (Additional file 1).

## Variables and instruments

The primary outcome of this aid-to-cessation trial will be between-group differences in continuous abstinence for the 26-week period between weeks 16 and 42. The primary outcome is measured at six months post-treatment discontinuation (week 42) rather than at treatment discontinuation (week 16), in line with the Russell Standard for international smoking cessation research [22]. To assess self-reported smoking habits, participants will complete a timeline followback (TLFB) at each contact visit. This measure has been shown to be accurate up to 12 months before interview [23]. Abstinence from smoking will be measured by self-report, exhaled carbon monoxide (CO_EXH_) < 10 ppm measured using a Micro+ Smokerlyzer (Bedfont Scientific) and salivary COT < 50 ng/L, which is a sufficient margin to allow for passive tobacco smoking. CO_EXH_ may be < 10 ppm in non-abstinent participants who have not smoked within 2 h [24]. Smoking cessation will be confirmed if CO_EXH_ levels are < 10 ppm and COT is < 50 ng/mL. Where participants self-report use of non-tobacco nicotine, CO_EXH_ will be used to confirm abstinence. Secondary outcomes will include the timing of the first lapse and first relapse, based on a dichotomous variable and defined as self-reported smoking seven days in a row at any time between two weeks after quitting and the trial end. Prolonged abstinence will also be reported and defined as any slips or lapses following the quit date [25].

At the baseline visit, the Fagerström Test for Nicotine Dependence and a smoking history questionnaire will be administered. The smoking history questionnaire will explore how long the participant has smoked for (e.g. age of first use, duration of use), their average consumption of cigarettes and the number of previous attempts to quit smoking. Data will also be collected regarding how determined they are to give up smoking on this attempt (on a Likert scale of 1–4) and to rate their chances of permanently quitting on this attempt (on a Likert scale of 1–6). The Minnesota Nicotine Withdrawal Scale will be administered to assess urge to smoke among other withdrawal symptoms, depressed mood, irritability, anxiety, difficulty concentrating, restlessness, increased appetite and sleep. Participants will also complete the Brief Questionnaire of Smoking Urges, which assesses cravings related to desire to smoke and expectations of positive effects anticipation of relief from negative affect. Demographic data, lifetime history of smoking (e.g. motivation, readiness to quit, previous attempts), level of social support, self-reported medical and psychiatric history will also be collected at baseline. A psychiatric diagnostic interview (SCID-5-RV), based on the DSM-5 will be conducted at baseline. The WHO ASSIST and AUDIT will be collected at baseline and visits at weeks 8 and 42. The Standardised Assessment of Personality – Abbreviated Scale (SAPAS), a brief screen for personality disorder, will be used at baseline. The WHO-5, Depression, Anxiety, and Stress Scale (DASS-21) and K10 will be administered at baseline and at weeks 8, 16 and 42 (Table 1). To optimise retention and adherence with the treatment protocols, telephone contact will be made with each participant once a fortnight beginning one week after baseline. Progress and adherence self-report data will be recorded on a questionnaire at these telephone calls. Participant reports of adverse effects will be recorded, appropriate interventions according to medical assessment will be implemented and AEs will be further monitored. Six months following the final study visit the participant will be contacted and a follow-up questionnaire conducted to assess if participants have continued to smoke, and at what level, since completing the trial. CO_EXH_ and COT measures will be taken.

**Table 1:** Summary of measures collected and other activities at each study visit

	Baseline	8 weeks	16 weeks	42 weeks (follow-up)
Demographic/health questionnaire	X			
Fagerström Test for Nicotine Dependence	X	X	X	X
Lifetime history of smoking	X			
SCID −5-RV	X			
SAPAS	X			
Exhaled CO (CO_EXH_)	X	X	X	X
MNWS	X	X	X	X
QSU-brief	X	X	X	X
Salivary cotinine	X	X	X	X
K10, DASS-21	X	X	X	X
AUDIT	X		X	X
WHO-5	X	X	X	X
WHO ASSIST	X		X	X
TLFB	X	X	X	
Dispensing of study medications	X	X		

Safety and tolerability outcomes will be assessed by comparison of reported AEs for all NAC-treated and placebo-treated participants and for subgroups. Subgroup analyses will be performed for groups categorised by variables including gender and SCID diagnosis. Participants will be considered to have completed the study if they completed all follow-up examinations to week 42. Individuals who never quit or who relapse (defined as smoking on seven consecutive days), will be examined separately as well as pooled as treatment failures. Non-cigarette tobacco use, but not non-tobacco nicotine use, will be categorised as smoking.

Intended use of bupropion, varenicline or NRT during the 42-week trial period will not be permitted but is only a withdrawal criterion if used during the 16-week treatment period. Some participants may decide to use NRT during the 42-week study. Although this is contrary to the directions given to participants in the study, it is not a study withdrawal criterion and these participants may remain in the study. COT levels will be measured using chromatographic techniques.

## Key outcome variables

### Data management and integrity

#### Confidentiality

Records of participation in this study will be held confidential so far as permitted by law. This will include de-identification of all documents relating to the trial by assigning a random number that will identify participants in terms of a dataset and not an individual. However, researchers, regulatory agencies and the Barwon Health Human Research Ethics Committee (HREC) will be able to inspect and have access to confidential data that identify participants by name.

#### Inter-rater reliability

The measures listed under ‘Variables and instruments’ in the study design section will be co-rated by trained trial clinicians. Inter-rater reliability will be checked before commencement of the trial and repeated every six months during the trial, using a standardised videotaped patient interview. Raters will need to achieve an intraclass (model 2,1) correlation score of 0.8 and will undergo further training as necessary during the study to maintain inter-rater reliability.

#### Sample size

The sample size was initially determined to be 360. In order to detect a 7% difference between the groups (with six-month smoking cessation, including participant attrition estimated at 1%), the sample size has been calculated for a Cochran-Mantel-Haenszel (CMH) test to acknowledge a cluster randomised design with three recruitment centres as strata (each with an overall six-month smoking cessation of 1% for placebo). A sample size of 180 in the NAC group and 180 in the control group (across three roughly equal size strata) achieves 80% power to detect an odds ratio (OR) = 7, assuming a two-sided type I error of 0.05. The sample size also achieves 80% power to detect a 7% difference in six-month smoking cessation between NAC and placebo groups assuming two-sided alpha = 0.05 and an 8% inflation of the required sample size due to clustering. However, the current trial has a recruitment target of 120 in total, with the intention of scaling up as more funding is acquired.

#### Statistical analysis

A modified ‘intention-to-treat’ approach, following CONSORT Statement guidelines, will be used. Missing values will be scrutinized to check for non-random distribution and analyses that utilize baseline data will be executed twice: once using observed data and once using multiple imputation under multivariate normal assumptions. The CMH test will be used to compare the proportions of relapse between the treatment groups (control versus intervention). The common OR and its 95% confidence interval will also be reported as well as the results of the Breslow-Day test for homogeneity of the ORs across the strata. In further supportive analyses, generalized linear mixed models (GLMMs) will be used to compare the rates of relapse after adjusting for baseline measurements and to explore possible interactions between baseline factors and the intervention. Average treatment group differences for the continuous secondary outcomes will be examined using a likelihood based mixed-effects model, repeated measures approach (MMRM). The MMRM models include the fixed categorical effects of treatment, visit and treatment-by-visit interaction, as well as the continuous fixed covariates of baseline score and baseline score-by-visit interaction. Effect sizes will be calculated using Cohen’s guidelines. All comparisons will be conducted using a two-sided alpha level of 0.05. There will be no interim analysis.

#### Study management and governance

Barwon Health is the sponsor of this study. The sponsor is indemnified for any harms arising from trial participation and it approves protocol amendments when ethics approval has been obtained. Principal investigators and trial clinicians manage the day-to-day running of the trial. Principal investigators are three well-established and experienced researchers in relevant fields. Itemised lists of various trial staff members are as follows:

#### Principal Investigators:

- Trial design
- Publication of study reports
- Study planning
- Provision of expertise to problem resolution
- Media promotion
- Funding application and management

#### Clinical Trial Coordinator:

- Case report form creation and management
- Research staff management
- Participant recruitment, contact and interviewing
- Liaison with external bodies (e.g. pharmacy, suppliers)
- Maintenance of trial equipment
- Training of research staff
- Organisation of research team meetings

There is no Data Management Committee for this trial due to the small sample size; however, independent data integrity will be assessed by independent researchers as required. Trial progress and processes are assessed every two weeks by the principal investigators at a research team meeting.

### Post-trial data provisions

A summary of the data will be provided to each participant along with whether they took the active or placebo medication. This information will be provided at the soonest reasonable opportunity after trial completion and will be provided electronically. The results will be published and disseminated throughout both formal and informal collegiate settings.

### Sharing of deidentified participant-level data

There is currently no plan to share participant-level data with external individuals.

## Conclusion

Tobacco smoking is a major preventable cause of morbidity and mortality. NAC may provide a safe, novel and effective treatment for smoking cessation, at low cost. The tolerability and safety of NAC as well as its efficacy in co-morbid psychiatric symptoms may have advantages in special populations compared to other pharmacotherapies for smoking cessation. If positive, of the use of NAC could be translated immediately into practice and established as a new treatment for smoking cessation. NAC additionally has a clearly elucidated potential mechanism of action in addiction. The planned trial, if positive, may result in substantial changes to the management of smoking with overt public health implications.

## Additional file

Additional file 1: SPIRIT 2013 Checklist: Recommended items to address in a clinical trial protocol and related documents. (DOCX 18 kb)

## Data Availability

Not applicable.
