# Supplementary material for: N-acetylcysteine for cessation of tobacco smoking: rationale and study protocol for a randomised controlled trial
Source: Trials. 2019 Sep 10;20:555. doi: 10.1186/s13063-019-3628-5 (PMC6734392; doi:10.1186/s13063-019-3628-5)
Supplement: Supplementary file 1 — SPIRIT 2013 Checklist: Recommended items to address in a clinical trial protocol and related documents. (DOCX 18 kb) [file 13063_2019_3628_MOESM1_ESM.docx]

| **TIMEPOINT** | **-1** | **T1** | **F1** | **F2** | **F3** |
| --- | --- | --- | --- | --- | --- |
|  | **Screening** | **Baseline/ Randomisation** | **Follow-Up**  **8 Weeks** | **Follow-Up**  **16 Weeks** | **Follow-Up**  **42 Weeks** |
| **ENROLMENT:** |  |  |  |  |  |
| Initial Phone Interview | X |  |  |  |  |
| Written Informed Consent Form |  | X |  |  |  |
| **INTERVENTION:** |  |  |  |  |  |
| N-acetylcysteine |  |  |  |  |  |
| **ASSESSMENTS:** |  |  |  |  |  |
| Demographic/health questionnaire |  | X |  |  |  |
| Fagerström Test for Nicotine Dependence |  | X | X | X | X |
| Lifetime history of smoking |  | X |  |  |  |
| SCID -5-RV |  | X |  |  |  |
| SAPAS |  | X |  |  |  |
| Exhaled CO (CO_EXH_) |  | X | X | X | X |
| MNWS |  | X | X | X | X |
| QSU-brief |  | X | X | X | X |
| Salivary cotinine |  | X | X | X | X |
| K10, DASS-21 |  | X | X | X | X |
| AUDIT |  | X |  | X | X |
| WHO-5 |  | X | X | X | X |
| WHO ASSIST |  | X |  | X | X |
| TLFB |  | X | X | X |  |
